# Comparison of parametric and machine methods for variable selection in simulated Genetic Analysis Workshop 19 data

**DOI:** 10.1186/s12919-016-0021-1

**Published:** 2016-10-18

**Authors:** Emily R. Holzinger, Silke Szymczak, James Malley, Elizabeth W. Pugh, Hua Ling, Sean Griffith, Peng Zhang, Qing Li, Cheryl D. Cropp, Joan E. Bailey-Wilson

**Affiliations:** 1 Center for Inherited Disease Research, IGM, Johns Hopkins School of Medicine, 333 Cassell Drive, Suite 1200, Baltimore, MD 21224 USA; 2 Computational and Statistical Genomics Branch, National Human Genome Research Institute, National Institutes of Health, 333 Cassell Drive, Suite 1200, Baltimore, MD 21224 USA; 3 Institute of Clinical Molecular Biology, Christian-Albrechts-University of Kiel, Christian-Albrechts-Platz 4, 24118 Kiel, Germany; 4 Division of Computational Bioscience, Center for Information Technology, National Institutes of Health, 9000 Rockville Pike, Building 12A, Bethesda, MD 20892 USA

## Abstract

Current findings from genetic studies of complex human traits often do not explain a large proportion of the estimated variation of these traits due to genetic factors. This could be, in part, due to overly stringent significance thresholds in traditional statistical methods, such as linear and logistic regression. Machine learning methods, such as Random Forests (RF), are an alternative approach to identify potentially interesting variants. One major issue with these methods is that there is no clear way to distinguish between probable true hits and noise variables based on the importance metric calculated. To this end, we are developing a method called the Relative Recurrency Variable Importance Metric (r2VIM), a RF-based variable selection method. Here, we apply r2VIM to the unrelated Genetic Analysis Workshop 19 data with simulated systolic blood pressure as the phenotype. We compare the number of “true” functional variants identified by r2VIM with those identified by linear regression analyses that use a Bonferroni correction to calculate a significance threshold. Our results show that r2VIM performed comparably to linear regression. Our findings are proof-of-concept for r2VIM, as it identifies a similar number of functional and nonfunctional variants as a more commonly used technique when the optimal importance score threshold is used.

## Background

Technology advances now allow high-throughput genetic data to be generated with ever-improving speed and affordability. One major bottleneck in utilizing this data is the development of bioinformatics tools that can identify true signals amongst the high level of noise. Since the rise in popularity of the genome-wide association study (GWAS) over a decade ago, thousands of variants have been identified that are associated with complex human traits, including pharmacological outcomes [1]. However, a large portion of the estimated heritability remains unexplained for many traits [2]. Machine learning methods are promising candidates to address this issue and are currently used in other scientific fields, including drug design [3].

One type of machine learning method is Random Forests (RF) [4]. A caveat to RF is that there is no standard method for selecting a set of variants with low levels of false positives while retaining adequate power. More commonly used parametric analyses, such as linear or logistic regression, produce statistics with generally accepted values for null error rates. However, due to factors such as multiple testing correction and correlated variables, these parametric thresholds may be too stringent and could result in a large number of false negatives. One way to obtain null error rates for machine methods is to generate empirical distributions by running thousands of permutation analyses. This is computationally impractical for genome-wide studies. We propose a more efficient method, which integrates different selection parameters to identify the appropriate threshold between signal and noise called the Relative Recurrency Variable Importance Metric (r2VIM). The ultimate goal of our project is to generate variant sets and prediction models to further our understanding and prediction of complex traits.

## Methods

### Variable selection

RF is a machine learning method that grows a collection of decision or regression trees to identify variables (e.g., single-nucleotide polymorphisms) that are associated with an outcome (e.g., blood pressure) while taking into account main and interaction effects. RF output is a ranked list of variables according to an importance score. Importance is calculated as the percent change in mean squared error after variable permutation. There is no standard method of selecting an importance score threshold that separates signal from noise. To this end, we incorporate and extend upon a previously proposed threshold selection method [5, 6]. Specifically, r2VIM combines 3 different variable selection components, as described below [6].

1. *Permutation-based importance score:* The raw variable importance metric (VIM) is calculated as the percent change in mean squared error (MSE) before and after random variable permutation.
2. *Estimate of null variance*: If no variables are associated with the trait, the VIMs should be symmetrically and randomly distributed around zero. In practice, the lowest VIM is usually negative. Thus, we use the absolute value of the lowest VIM as an estimate of the null variance [5, 6]. This estimate can be used as a threshold by selecting only those variables with VIMs greater than the null variance. In previous studies, we observed that this estimate alone may be too liberal for genome-wide data. To address this, we multiply the estimate by factors, or integers, to create more stringent thresholds [6]. For example, if the lowest VIM was-0.05, the null variance estimate would be 0.05. For more stringent thresholds, we could multiply by factors 2, 3, and 4 to get new thresholds of 0.10, 0.15, and 0.20, respectively.
3. *Recurrency*: Due to the inherent randomness of machines, variables that are deemed important in one run may disappear in a second run with a different random seed. Variables with high importance scores across runs are more likely to be true signals. In r2VIM, we run RF 5 to 10 times and select variables greater than the threshold factor from variable selection component (2) across runs.

For this analysis, we ran the above selection algorithm using RF on one of the simulated data sets for the systolic blood pressure (SBP) and Q1 (permuted) traits. We used the parallelizable Random Jungle (RJ) software to allow for the large number of input variables [7]. We ran RJ with regression trees (numeric inputs and outputs), 60,000 variables sampled at each node (mtry), and 4000 trees in the forest (ntree). These parameters were selected after testing several mtry/ntree combinations and selecting the one with the lowest prediction error. The analysis took a total of 5 h (1 h for each run). Each analysis was run using 64 cores (approximately 63 trees per node). Along with the genetic variants, we included sex, blood pressure medication, smoking status, and the top ten principal components (PCs) in the RJ analysis. Age was not included because of missingness (18/1937 with no age given). We calculated the variable relative importance score (RIS) for each run as *VIM/abs(min. VIM)*. This allows us to compare scores across runs. We combined the RIS values over the 5 runs by selecting the minimum value. This is considered “recurrency-corrected.”

We analyzed the same data set using linear regression. The model included main effect terms for the variant, blood pressure medication, smoking status, sex, age, and the top 10 PCs. Age was included in the linear regression analysis, as the inability to handle missingness is a weakness of r2VIM. Incorrect results would represent this weakness and be a fair comparison of the 2 methods. A Bonferroni correction on the *p* value for the variant term was used as the selection threshold. We compared the selected variants for the 2 methods based on the simulated disease model for this data.

### Data set

We used the Genetic Analysis Workshop 19 (GAW19) simulated SBP and Q1 phenotype data for 1937 unrelated individuals [8]. For both we used data set replicate numbered 100. The genetic data was generated using whole exome sequencing. Singletons and variants with any missingness were removed leaving 353,103 total variants. In the filtered set, 1047 variants were directly simulated to be functional, and 4328 variants are in genes with simulated functional variants. The functional variants had a wide range of effect sizes and minor allele frequencies, as described by the Genetic Analysis Workshop data contributors. This data set has previously undergone several quality filtering steps; however, an independent quality control analysis identified more samples that did not meet certain data quality requirements. A sibpair was identified by identity-by-descent (IBD) estimation analysis. The member of the pair with lower overall coverage and higher missingness rate was dropped. We also dropped 3 samples with 10× coverage of less than 0.7, 1 sample with missingness greater than 0.05, and 1 sample that was a clear outlier for the number of singletons. After these 5 samples were dropped, a PCs analysis was completed. The top 10 PCs were added as covariates into the linear regression model and were included as variables in the r2VIM analysis. The genetic data set is formatted as a standard PLINK binary input with genotypes coded as 0/1/2 indicating the number of alternative alleles present.

## Results

**Fig. 1:**
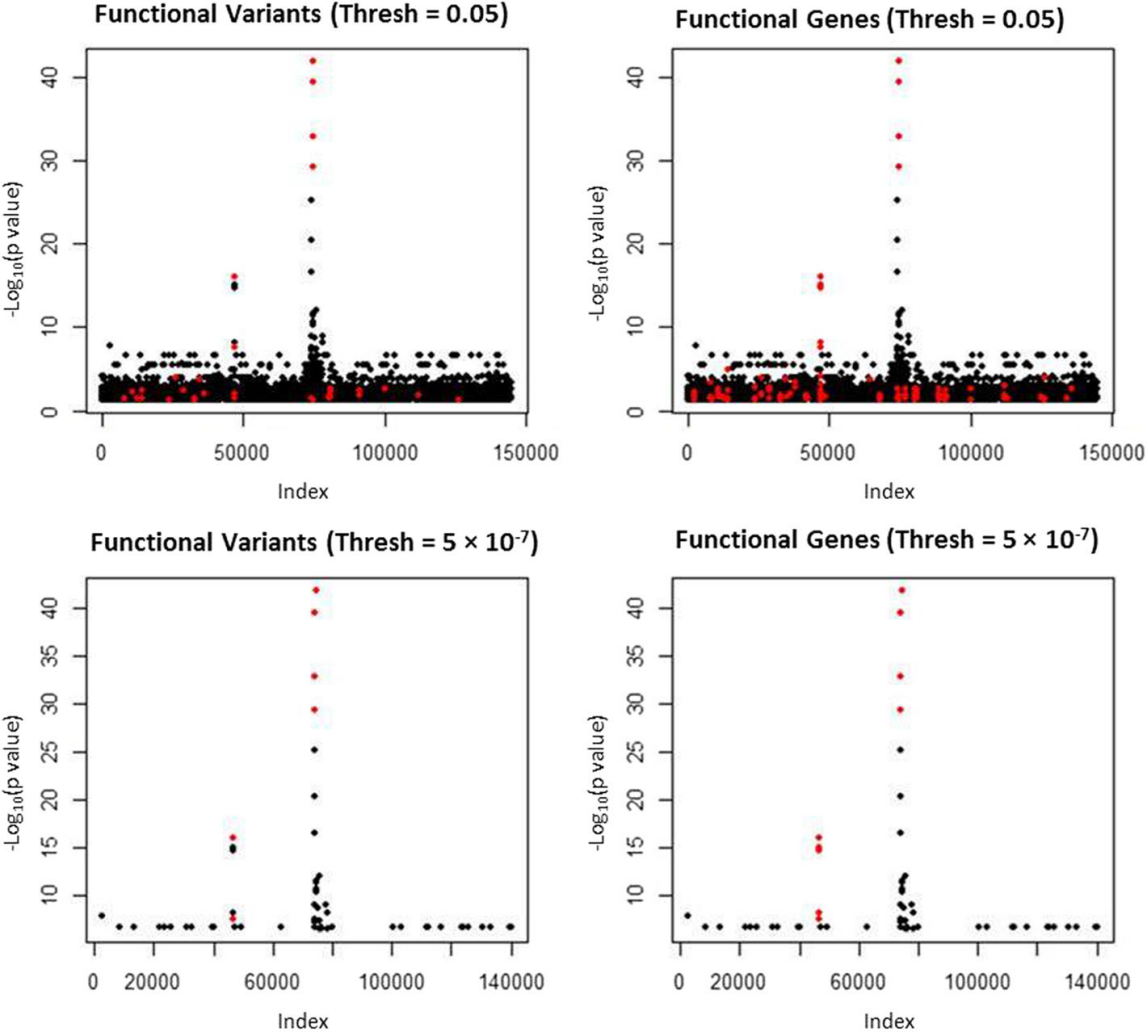
Results for the linear regression analysis of the simulated SBP phenotype for 2 *p* value thresholds (*p* <0.05 and *p* <5 × 10^−7^). The *x*-axis represents the variant index, which is in order of genome location. The *y*-axis shows the − log_10_(*p* value). The variants in *red* indicate functional variant (*left*) and variants in functional genes (*right*)

**Fig. 2:**
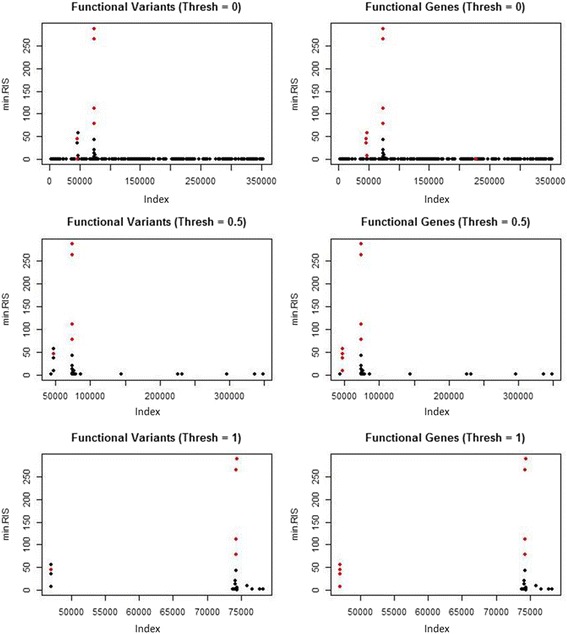
Results for the r2VIM analysis of the simulated SBP phenotype for 3 min.RIS thresholds (RIS <0, RIS <0.5, and RIS <1). The *x*-axis represents the variant index, which is in order of genome location. The *y*-axis shows the min.RIS. The variants in *red* indicate functional variant (*left*) and variants in functional genes (*right*)

We ran r2VIM and linear regression on the GAW19 SBP simulated phenotype. Figure 1 shows the results for the linear regression analysis of SBP. Each row represents a different *p* value selection threshold: *p* <0.05 and *p* <5 × 10^−7^ (Bonferroni corrected). All of the variants selected at the given thresholds are shown in black (not simulated to be functional) and red (functional). The left column shows the functional variants as red, and the right column shows the variants in functional genes as red. The counts for the selected variants are shown in Table 1. Figure 2 shows the results for the r2VIM analysis. Here we show the selected variants at 3 minimum RIS thresholds: min.RIS <0, min.RIS <0.5, and min.RIS <1. The counts for the total functional versus nonfunctional variants selected are shown in Table 1.

**Table 1:** Counts for the number of variants selected at different thresholds for linear regression (*p* value) and r2VIM (min.RIS)

Method	Threshold	All (~350 k)	Func. vars. (1047)	Func. genes (4328)
Linear Regression	*p* <0.05	7739	35	136
	*p* <5 × 10^−7^	59	6	9
r2VIM	min.RIS >0	340	6	9
	min.RIS >0.5	37	5	8
	min.RIS >1	25	5	8

The total number selected, the number of variants simulated directly to be functional, and the number of variants in the functional genes are shown

**Fig. 3:**
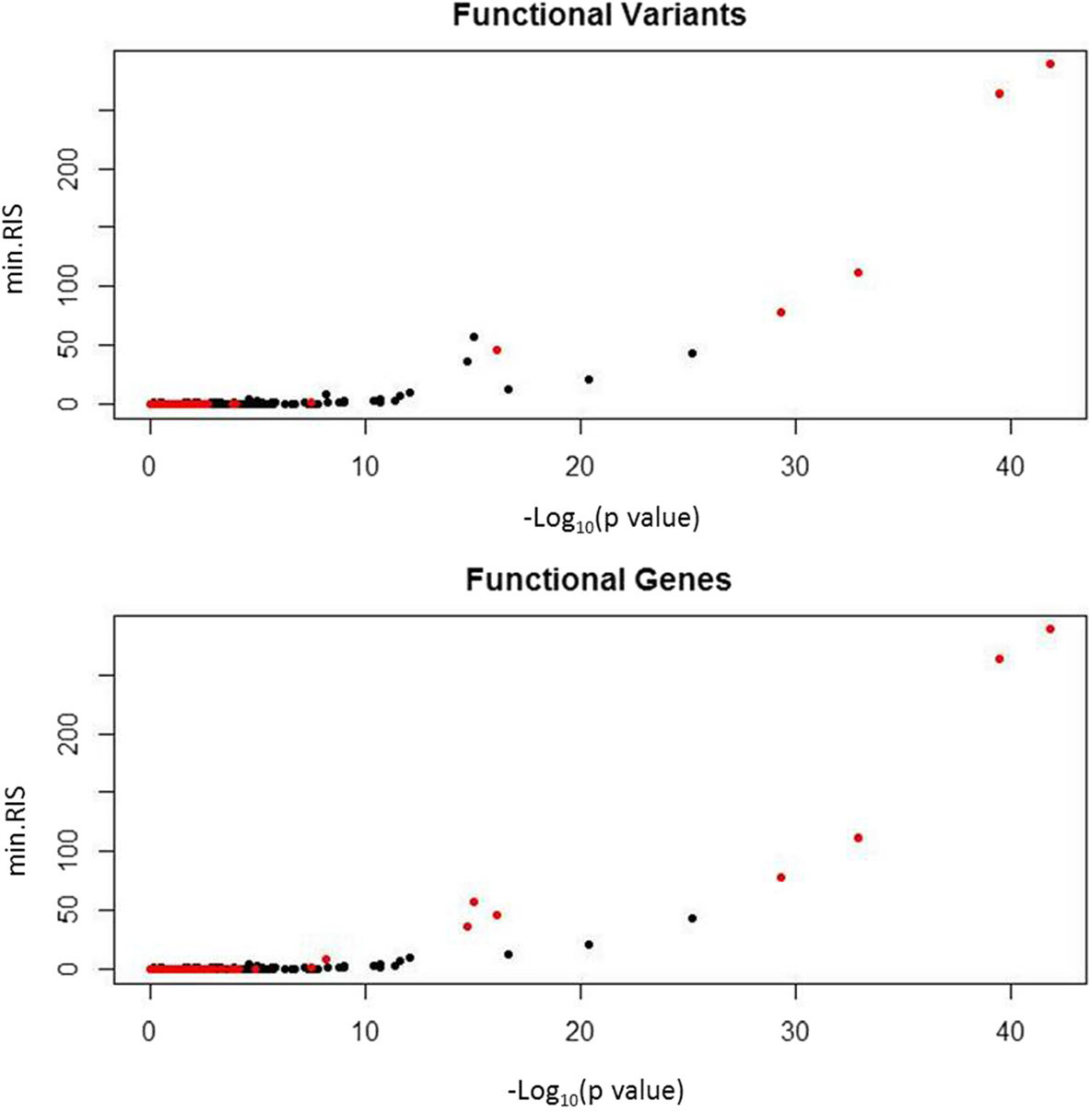
Comparison of the min.RIS score from the r2VIM analysis (y-axis) and the − log_10_(*p* value) for the linear regression analysis of the simulated SBP phenotype. The variants in *red* indicate functional variant (*top*) and variants in functional genes (*bottom*)

Next, we assessed the correlation between the *p* values and the minimum RIS scores for linear regression and r2VIM, respectively (Fig. 3). These graphs suggest agreement between the scores. It does not appear that functional variants were identified as having strong signals in one method and not the other. Of note, variants in black are not necessarily nonfunctional, as they could still be in linkage disequilibrium with a functional variant but not in the same gene. This makes it difficult to do a reliable true-positive versus false-positive assessment.

**Fig. 4:**
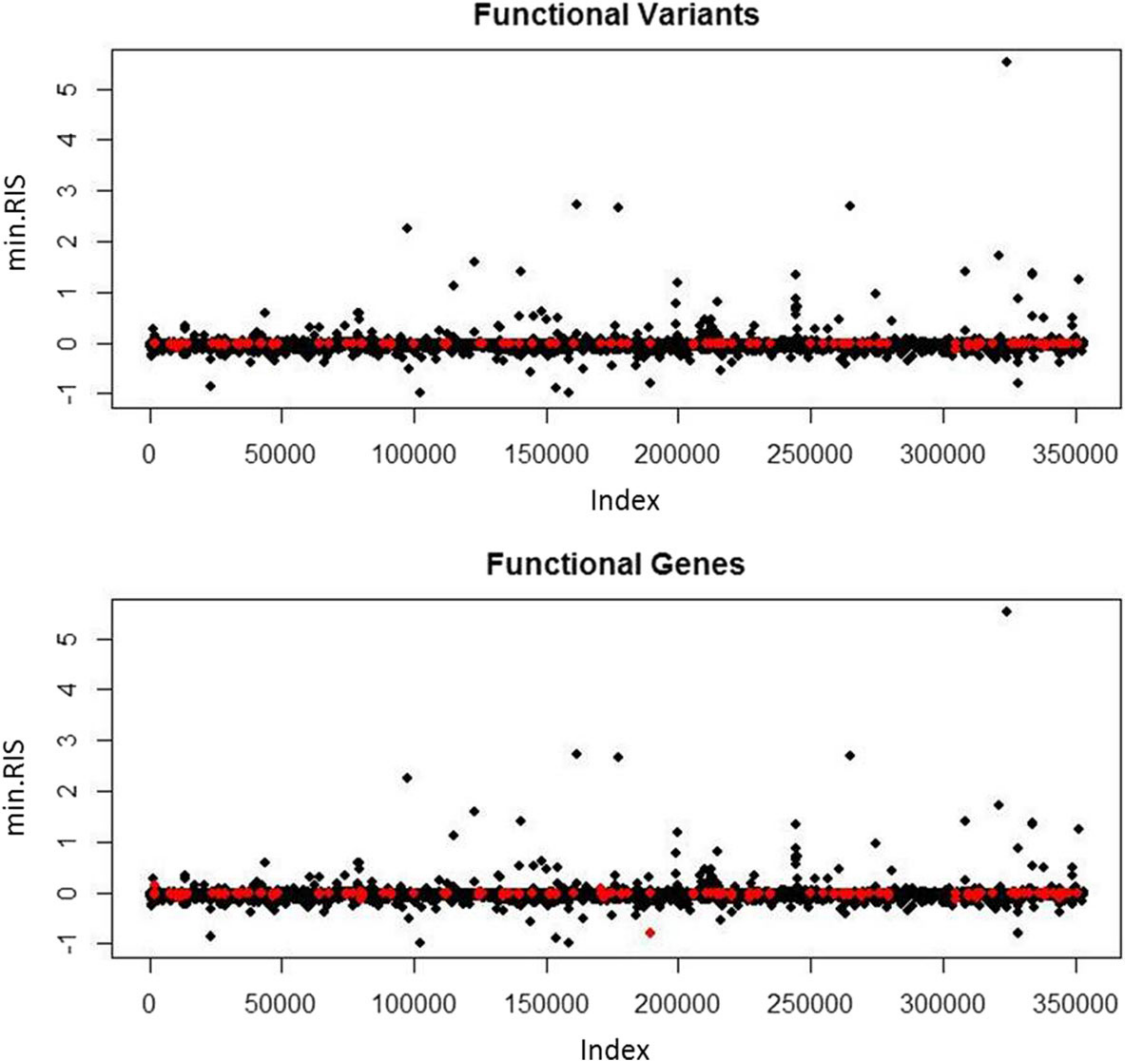
Results for the r2VIM analysis of the simulated Q1 phenotype. The *x*-axis represents the variant index, which is in order of genome location. The *y*-axis shows the min.RIS. The variants in *red* indicate functional variant (*left*) and variants in functional genes (*right*) from the SBP phenotype simulated model

Finally, we ran r2VIM using the Q1 phenotype (Fig. 4). This phenotype was not simulated to be correlated with any of the variants. Therefore, any positive associations should represent false-positive selection. None of the variants were significant after Bonferroni correction for the linear regression analysis of the Q1 data set (results not shown). Variants simulated to be functional in the SBP phenotype model are shown in red. Certain variants in this model have high min.RIS scores; however, this could be an artifact of the simulation method and further testing needs to be done.

## Discussion

For this GAW19 data set, linear regression and r2VIM had similar true-positive and false-positive selection counts when a Bonferroni corrected *p* value and a minimum RIS score of 0.5 were used as thresholds. Both methods identified the 2 genes with the strongest simulated effects, as shown by the 2 highest peaks in Figs. 1 and 2. Although a key motivation for r2VIM development is the identification of more complex models (e.g., interactions with small main effects), this is an important proof-of-concept for main-effect-only selection, as these are likely to be present along with interaction effects. Notably, neither method identified a large proportion of the functional variants. Both appeared to have an effect size/allele frequency threshold that resulted in no power for selection. Future r2VIM testing will be done using techniques to boost power for variants, such as binning by functional region.

There are several major limitations of RF (and, therefore, r2VIM) including the inability to handle any data missingness. In this analysis, we dropped variants with missing data points. Imputation is another option; however, it is often time-consuming and can result in many variants with unreliable calls. Future work will be done on the best and most efficient way to impute genotypes for r2VIM analysis to balance data gain with data quality. Another current limitation is the amount of computational power required to run RJ on large data sets. This analysis required a large number of high-memory processors. Future work will be done on ways to improve memory consumption. Finally, the best way to select the RIS threshold is still not clear. Here we showed 3 different thresholds and selected the best one according to the simulated model. This threshold selection process would not be possible in a biological data set where the underlying model is unknown. However, the optimal threshold is going to be dependent on the underlying model and will be different for different data sets. To this end, we plan on incorporating a “null distribution” analysis, by permuting the phenotype and running r2VIM a computationally feasible number of times. By comparing this distribution to the alternate, we may be able to determine a more regimented threshold selection process.

## Conclusions

For this analysis, we were able to show that r2VIM is a promising candidate for variable selection, as it performs as well as the more commonly used linear regression method for the identification of main effects. Power to detect causal variants was low as expected given the simulated model, but false positive rates were similar between linear regression and RF for the SBP trait. Perhaps the most important model to simulate will be one that contains main and interactions effects, as true biology is likely to contain both. The final goal is to generate predictive models that allow for all types of effects, which would require a method robust to more than just main effects. With these models, we will be able to gain deeper insight into the true etiology of complex human disease.

## References

[1] Welter D, MacArthur J, Morales J, Burdett T, Hall P, Junkins H (2013). The NHGRI GWAS Catalog, a curated resource of SNP-trait associations. Nucleic Acids Res.

[2] Manolio TA, Collins FS, Cox NJ, Goldstein DB, Hindorff LA, Hunter DJ (2009). Finding the missing heritability of complex diseases. Nature.

[3] Gertrudes JC, Maltarollo VG, Silva RA, Oliveira PR, Honório KM, da Silva AB (2012). Machine learning techniques and drug design. Curr Med Chem.

[4] Cutler A, Cutler DR, Stevens JR, Zhang C, Ma Y (2012). Random Forests. Ensemble machine learning.

[5] Strobl C, Malley J, Tutz G (2009). An introduction to recursive partitioning: rationale, application, and characteristics of classification and regression trees, bagging, and random forests. Psychol Methods.

[6] Szymczak S, Holzinger E, Dasgupta A, Malley J, Molloy A, Mills J (2015). r2VIM: a new variable selection method for random forests in genome-wide association studies. Pers Commun.

[7] Schwarz DF, Konig IR, Ziegler A (2010). On safari to Random Jungle: a fast implementation of Random Forests for high-dimensional data. Bioinformatics.

[8] Blangero J, Teslovich TM, Sim X, Almeida MA, Jun G, Dyer TD (2015). Omics-squared: Human genomic, transcriptomic and phenotypic data for Genetic Analysis Workshop 19. BMC Proc.

